# Statistical Predictors of Psychosocial Impairment in Body-Focused Repetitive Behaviors

**DOI:** 10.1017/S1092852921000468

**Published:** 2021-04-30

**Authors:** Jon E. Grant, Ibrahim H. Aslan, Samuel R. Chamberlain

**Affiliations:** a Department of Psychiatry & Behavioral Neuroscience, University of Chicago, Chicago, IL, USA; b Department of Psychiatry, University of Southampton, Southampton, UK; c Department of Psychiatry, University of Cambridge & Cambridgeshire/Peterborough NHS Foundation Trust, UK

**Keywords:** trichotillomania, skin picking, disability, dysfunction, impairment, predictors

## Abstract

**Objective:**

Skin picking disorder and trichotillomania, also characterized as body-focused repetitive behaviors (BFRBs), often lead to functional impairment. Some people with BFRBs, however, report little if any psychosocial dysfunction. There has been limited research as to which clinical aspects of BFRBs are associated with varying degrees of functional impairment.

**Methods:**

Adults (n=98), ages 18-65 with a current diagnosis of trichotillomania (n=37), skin picking disorder (n=32), trichotillomania plus skin picking disorder (n=10), and controls (n=19) were enrolled. Partial least squares regression (PLS) was used to identify variables associated with impairment on the Sheehan Disability Scale.

**Results:**

PLS identified an optimal model accounting for 45.8% of variation in disability. Disability was significantly related to (in order of descending coefficient size): severity of picking, perceived stress, comorbid disorders (specifically, anxiety disorders / obsessive-compulsive disorder), trait impulsivity, family history of alcohol use disorder, atypical pulling/picking sites, and older age.

**Conclusions:**

At present mental disorders are viewed as unitary entities; however, the extent of impairment varies markedly across patients with BFRBs. These data suggest that whereas symptom nature/severity is important in determining impairment, so too are other variables commonly unmeasured in clinical practice. Outcomes for patients may thus be maximized by rigorously addressing comorbid disorders; as well as integrating components designed to enhance top-down control and stress management. Interestingly, focused picking and emotional pulling were linked to worse disability, hinting at some differences between the two types of BFRBs, in terms of determinants of impairment.

## Introduction

Skin picking disorder and trichotillomania are characterized, respectively, by recurrent skin picking and hair pulling, resulting in excoriations or hair loss and functional impairment.^1^ Due to phenomenological similarities between these two disorders, they have further been labeled as body-focused repetitive behaviors (BFRBs).^2^ As with most psychiatric disorders, BFRB severity runs from mild to severe, with multiple studies examining what factors predict symptom severity.^3–5^ Degrees of functional impairment associated with BFRBs also appear to run the gamut from no impairment to grossly dysfunctional, but there has been little examination of which variables account for this wide-ranging degree of impairment seen in individuals with these behaviors.

In the small amount of research done in BFRBs, we see that impairment in both adults and children may be related to symptom severity,^3, 6,7^ to co-occurring depressive and anxiety symptoms,^4,8^ and older age of BFRB onset.^9^ These previous studies have been somewhat limited by examination of a relatively narrow range of variables – for example, considering the impact of depression and anxiety but not impulsive traits or presence of other comorbid symptoms.

When we look to other mental health disorders, we find some additional understanding of what predicts functional difficulties. Several studies examining predictors of functional impairment in obsessive compulsive disorder (OCD) have found that symptom severity and co-occurring disorders are associated with greater levels of functional impairment.^10–13^ In a large study of individuals with bipolar disorder (N=469), the co-occurrence of ADHD or any anxiety disorder predicted poorer psychosocial outcomes.^14^ In a study of body dysmorphic disordered (BDD) (n=256), Marques and colleagues found that symptom severity and comorbid depression were significantly associated with psychosocial disability.^15^ Taken together, research suggests that symptom severity and comorbidity appear to be the most likely predictors of who with a disorder struggles with greater functional disability.

The present study sought to examine statistical predictors of functional impairment in adults with BFRBs who were assessed with a range of clinical and psychological measures. Based on the extant literature in other disorders, we predicted that BFRB symptom severity and co-occurring comorbidities would be associated with greater functional impairment.

## Methods

### Participants

Non-treatment seeking adults (n=98), aged 18-65 years, with a current DSM-5 diagnosis of trichotillomania (n=37), skin picking disorder (n=32), both trichotillomania and skin picking disorder (n=10), and controls (n=19) were enrolled. Participants were recruited from March 2017 to September 2018 at a university academic medical center in an urban area. Exclusion criteria included: changes in psychotropic medication three months prior to study entry (for those participants taking medications), or any medical condition that would preclude a person’s ability to complete questionnaires. Additionally, controls, who were age and gender-matched, were excluded if they had any current or lifetime psychiatric disorder or any current or lifetime use of psychotropic medication.

The Institutional Review Board at the University of Chicago approved the study and consent forms. After participants were given a thorough explanation of study procedures and an opportunity to ask any questions, they provided written informed consent. This research was conducted in accordance with the principles of the Declaration of Helsinki.

### Assessments

Demographic variables such as age and gender were collected on each participant. All participants also completed the Mini International Neuropsychiatric Interview 7.0 (MINI 7.0), as well as diagnostic modules for trichotillomania, and skin picking disorder, as well as BFRB symptom measures.^16^

To examine psychosocial impairment, we used the Sheehan Disability Scale (SDS).^17^ Additionally, participants completed a semi-structured interview assessing the clinical characteristics of BFRBs, as well as personal and family medical and psychiatric histories. Other measures assessed anxiety, depression, impulsivity, and triggers to pull or pick. These measures included the following: Milwaukee Inventory for Subtypes of Trichotillomania-Adult Version (MIST-A),^18^ which assesses intentionality and emotionality (I and E subscales); Massachusetts General Hospital Hair Pulling Scale (MGH-HPS),^19^ a severity scale of hair pulling; Milwaukee Inventory for the Dimensions of Skin Picking (MIDAS),^20^ which examines automatic and focused picking styles (A and F subscales); Skin Picking Scale-Revised (SPS-R),^21^ a scale of picking severity; the Perceived Stress Scale (PSS)^22^ and the Barratt Impulsivity Scale (BIS-11, total score).^23–25^

### Data Analysis

We used Partial Least Squares regression (PLS)^26–28^ to identify measures associated with disability (SDS total scores) across the whole sample. PLS is a multivariate, iterative technique that constructs one or more latent variables that optimally explain relationships between two matrices. Here the X matrix was the BFRB clinical measures of interest, and the Y matrix was the disability scores. The following explanatory variables of interest were included in the initial PLS model: age, duration of illness, gender, family history (of trichotillomania, skin picking disorder, or alcohol use disorder in first-degree relatives), MGH total scores, SPS-R total scores, PSS total scores, MIST-A I and E scores, MIDAS A and F scores, nature and frequency of picking sites (face, scalp, body, or other), presence of current anxiety disorder, presence of current depressive episode, presence of current OCD, and past year alcohol/substance use disorder. We used PLS because conventional regression models are unsuitable in situations involving inter-correlated variables, as was expected to be the case in this study. Analysis was conducted using JMP Pro Software, with automatic unbiased imputation for missing data. The PLS modelling was fitted using a non-linear iterative partial least squares (NIPALS) algorithm. Per convention, explanatory measures with a Variable Importance Parameter (VIP) of >0.8 were retained in the final PLS model – this is a means of screening out and removing statistically unimportant variables (i.e. those unrelated to disability).

## Results

The sample comprised 98 individuals, of whom 37 had trichotillomania, 32 skin picking disorder, 10 had both, and 19 were controls. An overview of the characteristics of each group is presented in Table 1. The entire sample’s mean age was 30.1 (8.4) years, and 82 (84.5%) were female and 75 (76.5%) were white Caucasian.

Of the clinical subjects, the mean (SD) SDS total score for trichotillomania was 7.5 (6.7), for skin picking disorder it was 9.2 (8.9), and for those with both trichotillomania and skin picking it was 12.0 (8.2). SDS scores in healthy controls were 0.3 (1.3). The groups differed on SDS scores overall (F=7.0865, p<0.001). Each clinical group was significantly impaired versus the controls (each p≤0.001) based on post hoc t-tests but clinical groups did not differ significantly from each other (each p>0.10).

**Fig. 1:**
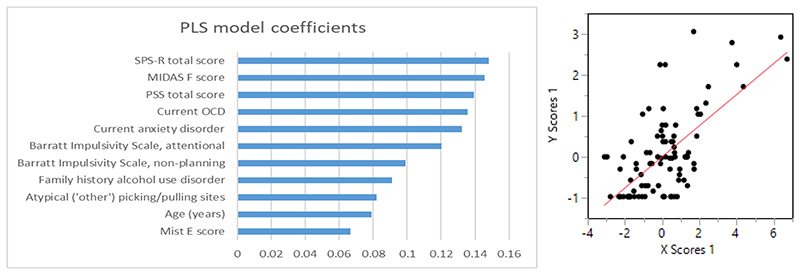
Optimal PLS model listing variables statistically explaining disability in the sample. The left panel shows the model coefficients, ranked from high to low in terms of coefficient magnitude, and the right graph shows the plot of latent variable scores.

The initial PLS model identified a one-factor model that minimised the PRESS statistic, and accounted for 14.5% of variance in the explanatory parameters, and 45.8% of variation in disability. Retention of those explanatory variables with VIP >0.8 generated yielded a one-factor final model accounting for 24.8% of variation in the explanatory parameters and 45.8% of variation in disability. The variables in the final model are shown in Figure 1.

## Discussion

In this study, we examined a wide range of clinical variables in BFRBs to see which most strongly predicted whether a person would be psychosocially impaired due to their BFRB. Both trichotillomania and skin picking disorder were associated with significantly greater psychosocial impairment compared to controls but both disorders were, on average, associated with only mild to moderate impairment based on the SDS. Of the variables that predicted worse psychosocial impairment, skin picking severity, focused picking, and greater perceived stress were the strongest variables associated with impairment. Partly contrary to both our hypothesis and to previous literature,^4,9^ greater dysfunction was not associated with worse total trichotillomania symptom severity as indexed by the MGH-HPS, or with current depressive disorder. Because frequent picking often results in noticeable scarring, the finding that severity of picking is associated with greater likelihood of impairment is not surprising. The lack of an equivalent finding in terms of hair pulling severity may speak to a greater ability to hide the consequences of pulling (e.g., redistributing hair to cover bald patches) and/or speak to some other variable suggestive of greater resilience in people who pull compared to those who pick. Also relevant in interpreting this finding is that higher scores on the MIST-A E measure were associated with worse impairment, even if MGH-HPS was not, suggesting that hair pulling in response to emotions (which could be seen as a sub-score of severity) is linked to higher disability.

The other important finding from this study is that impulsivity (at least two domains from the Barratt being attentional and non-planning impulsivity) and family history of alcoholism are all strongly associated with greater impairment in BFRBs. Taken together, these findings suggest that at least some people with BFRBs have a personal and familial link to impulsivity, which is in keeping with a recent subtyping analysis of BFRBs.^29^ This feature of impulsivity seems to be a strong predictor of psychosocial impairment, even when symptom severity is not a strong predictor, as in trichotillomania symptoms. Thus, this suggests that treatment of BFRBs may need to address the impulsivity, either through psychotherapy or medication, in those who are more impaired by their BFRB.

This study, while demonstrating some novel findings, also has some important limitations. First, there may be multiple ways to assess psychosocial impairment. As a self-report measure, the SDS has the limitation that people may under-report the extent of their BFRB’s influence on their functioning. Second, the clinical variables were all reported by the participant without any corroboration from others. Third, as a cross-sectional study, these findings may not provide a clear temporal relationship between impairment and these clinical variables. Fourth, an exploration of many possible predictors may result in chance findings. Thus, replication of these results in an independent sample would be particularly important. Finally, we recognize that disability and impairment are not the same constructs. However, as SDS captures disability relating to three generally important life domains, for the purposes of this paper we refer to it as capturing impairment.

In conclusion, this study indicates that a considerable proportion of functional impairment in BFRBs is accounted for by variables other than severity per se – though severity is also important. Cohesive clinical understanding of BFRBs, and treatment thereof, is likely to require incorporation of a variety of scales not only relating to the core symptoms themselves. It is also worthwhile to consider how these findings may impact treatment approaches and future research. In terms of the clinical importance of these findings, these data suggest that not everyone with a BFRB should be treated identically. A thorough assessment of functionality may suggest that certain people with BFRBs may need more tailored treatment approaches, such as adding elements of dialectical behavior therapy for coping skills enhancement, behavioral techniques to reduce impulsivity, or even pharmacotherapy to reduce impulsivity. Additionally, these data suggest that even though skin picking and trichotillomania are both BFRBs, they should not always be treated in the same fashion. Impairment may be particularly important to discuss with those people who have a primary skin picking disorder. Additionally, if skin picking severity is more likely associated with impairment, clinicians may want to consider more intensive therapy, with a focus on functionality, for people with skin picking, and even enlist the help of dermatologists or primary doctors to see what can be done to help the healing of excoriations. These suggestions lack empirical support, however, and perhaps frame one direction for future research such as understanding whether interventions may need to differ based on functional impairment.

## Figures and Tables

**Table 1:** Demographic and Clinical Characteristics of the Participants.

	Mean (SD) or N [%]			
	Trichotillomania [n=37]	Skin Picking Disorder [n=32]	Trichotillomania + Skin picking Disorder [n=10]	Non-affected controls [n=19]
Age	31.3 (8.1)	31.5 (9.9)	28.1 (7.6)	26.2 (8.5)
Age of onset	11.6 (4.6)	13 (8.7)	11.3 (3.9)	-
Duration of illness	19.5 (10.3)	18.5 (11.7)	16.8 (8.7)	-
Sex Female/Male	34/3	29/3	9/1	15/4
Family history*	24 [65]	22 [69]	8 [80]	5 [26]
SDS	7.5 (6.7)	9.2 (8.9)	12.0 (8.2)	0.3 (1.3)
MGH-HPS	16.5 (4.4)	2.3 (4.7)	12.6 (5.9)	0.0 (0.0)
SPS-R	3.3 (4.3)	15.9 (5.4)	11.9 (3.6)	0.8 (2.4)
MIST-A I	31.1 (13.0)	34.9 (7.9)	34 (8.7)	36.6 (2.4)
MIST-A E	20.8 (8.3)	3.8 (9.2)	23.9 (10.4)	0.3 (1.4)
MIDAS-A	13.7 (3.5)	18.8 (4.0)	16.5 (5.2)	14.6 (2.9)
MIDAS-F	7.7 (4.7)	18.4 (5.6)	17.3 (6.4)	4.8 (4.5)
PSS	20.1 (6.8)	20.3 (7.5)	22.1 (4.4)	11.5 (5.5)
BIS11	67.3 (15.0)	63.6 (18.9)	64.0 (25.0)	64.6 (8.9)
Current comorbid conditions n [%]				
Major depressive disorder	2 [5.4]	1 [3.1]	1 [10]	0 [0]
Any anxiety disorder	8 [21.6]	3 [9.4]	2 [20]	0 [0]
OCD	0 [0]	2 [6.3]	1 [10]	0 [0]
ADHD	0 [0]	2 [6.3]	1 [10]	0 [0]
Any substance use disorder	2 [5.4]	2 [6.3]	0 [0]	0 [0]

SDS = Sheehan Disability Scale; MGH-HPS= Massachusetts General Hospital Hairpulling Scale; SPS-R= Skin Picking Scale-Revised; MIST-A I/E = Milwaukee Inventory for Subtypes of Trichotillomania-Adult Version Intention and Emotion scores; MIDAS-A/F = Milwaukee Inventory for Dimensions of Picking Automatic and Focused scores; PSS = Perceived Stress Scale; BIS11= Barratt Impulsiveness Scale 11; OCD = obsessive-compulsive disorder; ADHD= attention-deficit hyperactivity disorder.

* Family history of trichotillomania, skin picking disorder, or alcohol use disorder in a first-degree relative
